# PD-1/PD-L1 combined with LAG3 is associated with clinical activity of immune checkpoint inhibitors in metastatic primary pulmonary lymphoepithelioma-like carcinoma

**DOI:** 10.3389/fimmu.2022.951817

**Published:** 2022-10-03

**Authors:** Yu-Min Zhong, Kai Yin, Yu Chen, Zhi Xie, Zhi-Yi Lv, Jin-Ji Yang, Xue-Ning Yang, Qing Zhou, Bin-Chao Wang, Wen-Zhao Zhong, Ling-Ling Gao, Wen-Bin Zhou, Ji Chen, Hai-Yan Tu, Ri-Qiang Liao, Dong-Kun Zhang, Shui-Lian Zhang, Dan-Xia Lu, Hong-Bo Zheng, Heng-Hui Zhang, Yi-Long Wu, Xu-Chao Zhang

**Affiliations:** ^1^ Guangdong Cardiovascular Institute, Guangdong Provincial People’s Hospital, Guangdong Academy of Medical Sciences, Guangzhou, China; ^2^ Guangdong Lung Cancer Institute, Guangdong Provincial Key Laboratory of Translational Medicine in Lung Cancer, Cancer Center, Guangdong Provincial People’s Hospital, Guangdong Academy of Medical Sciences, Guangzhou, China; ^3^ Medical Research Center, Guangdong Provincial People’s Hospital, Guangdong Academy of Medical Sciences, Guangzhou, China; ^4^ The Second School of Clinical Medicine, Southern Medical University, Guangzhou, China; ^5^ Department of Thoracic Surgery, Guangdong Provincial People’s Hospital, Guangdong Academy of Medical Sciences, Guangzhou, China; ^6^ Department of Medical Affairs, Genecast Biotechnology, Wuxi, China

**Keywords:** Lymphoepithelioma-like carcinoma, PD-1/PD-L1 inhibitors, PD-1, PD-L1, LAG3

## Abstract

Primary pulmonary lymphoepithelioma-like carcinoma (PLELC) is an Epstein–Barr virus (EBV)-related, rare subtype of non-small-cell lung cancer (NSCLC). Immune checkpoint inhibitors (ICI) show durable responses in advanced NSCLC. However, their effects and predictive biomarkers in PLELC remain poorly understood. We retrospectively analyzed the data of 48 metastatic PLELC patients treated with ICI. Pretreated paraffin-embedded specimens (n = 19) were stained for PD-1, PD-L1, LAG3, TIM3, CD3, CD4, CD8, CD68, FOXP3, and cytokeratin (CK) by multiple immunohistochemistry (mIHC). Next-generation sequencing was performed for 33 PLELC samples. Among patients treated with ICI monotherapy (n = 30), the objective response rate (ORR), disease control rate (DCR), median progression-free survival (mPFS), and overall survival (mOS) were 13.3%, 80.0%, 7.7 months, and 24.9 months, respectively. Patients with PD-L1 ≥1% showed a longer PFS (8.4 *vs*. 2.1 months, *p* = 0.015) relative to those with PD-L1 <1%. Among patients treated with ICI combination therapy (n = 18), ORR, DCR, mPFS, and mOS were 27.8%, 100.0%, 10.1 months, and 19.7 months, respectively. Patients with PD-L1 ≥1% showed a significantly superior OS than those with PD-L1 <1% (NA versus 11.7 months, *p* = 0.001). Among the 19 mIHC patients, those with high PD-1/PD-L1 and LAG3 expression showed a longer PFS (19.0 *vs*. 3.9 months, *p* = 0.003). ICI also showed promising efficacy for treating metastatic PLELC. PD-L1 may be both predictive of ICI treatment efficacy and prognostic for survival in PLELC. PD-1/PD-L1 combined with LAG3 may serve as a predictor of ICI treatment effectiveness in PLELC. Larger and prospective trials are warranted to validate both ICI activity and predictive biomarkers in PLELC.

This study was partly presented as a poster at the IASLC 20th World Conference on Lung Cancer 2019, 7–10 September 2019, Barcelona, Spain.

## Introduction

Lymphoepithelioma-like carcinoma (LELC), an Epstein–Barr virus (EBV)-associated malignancy, is an undifferentiated carcinoma of epithelial cells showing frequent prevalence in the nasopharynx (1–3). Pulmonary LELC (PLELC) is a rare subtype of non-small-cell lung cancer (NSCLC) that was first described in 1987 and is mostly reported in Asian countries (4–8). Since then, nearly 1,500 cases have been reported in the literature (1, 3, 7). The rates of EGFR mutation and ALK rearrangement are very low in PLELC and targeted therapies show limited efficacy among these patients (7, 9, 10). Palliative chemotherapy and radiotherapy remain the main treatment options for metastatic primary PLELC, but have limited efficacy (11, 12). Although several efforts have been made during the past few years, the standard treatment strategy for metastatic PLELC remains controversial and multidisciplinary management has been recommended for this disease (1, 2, 5, 7, 12). Emerging treatment approaches, including immune therapies, may be an option for improving the outcome of advanced PLELC.

Immune checkpoint inhibitors (ICI), particularly programmed cell death protein-1 (PD-1)/programmed cell death-ligand 1 (PD-L1) inhibitors, are standard treatment options for several malignancies, including lung cancer (13–16). Several PD-1/PD-L1 inhibitors, including pembrolizumab, nivolumab, atezolizumab, and camrelizumab, have been approved for treating advanced NSCLC (17–20). However, the role of ICI in advanced PLELC remains unclear. Several retrospective studies suggest a favorable response to PD-1/PD-L1 inhibitors in patients with PLELC (2, 3, 21, 22). Moreover, ICIs are effective in other virus-related malignancies like EBV-associated gastric cancer and nasopharyngeal carcinoma. These data necessitate further study on the impact of ICI on EBV-associated PLELC (23–25).

Preliminary data show that the immune system plays a critical role in the emergence of PLELC. Several studies confirm the high expression of PD-L1 in PLELC (9, 26, 27). Moreover, the tumor microenvironment (TME) of EBV-positive malignancies shows abundant tumor-infiltrating lymphocytes (TILs) and PD-L1 overexpression, classified as the immune-inflamed phenotype (28–30). These immune characteristics of the TME may reduce the benefit of ICI in PLELC. Gaining more insight into the immunological aspect of TME is important for identifying biomarkers for ICI therapeutic efficacy and developing novel combinational immunotherapy for this unique cancer type.

Therefore, we aimed to examine the correlation between immunogenic and genomic features and the responses of ICI in a relatively large PLELC cohort.

## Materials and methods

### Patient characteristics

We retrospectively reviewed the medical records of patients who were diagnosed with primary PLELC at the Guangdong Lung Cancer Institute (GLCI) between January 2016 and May 2021. Forty-eight metastatic PLELC patients who underwent ICI treatment were analyzed. The electronic records of these patients were reviewed, including their clinicopathological features, available gene mutation data, and ICI treatment outcomes. PLELC was diagnosed based on the criteria of the World Health Organization (31). We conducted nasopharyngoscopy or nasopharyngeal radiological imaging to rule out metastasis from LELC. This retrospective study adhered to the rules and principles of clinical studies for protecting human subjects. The pathological tumor stage was modified according to the Eighth Edition of the American Joint Committee on Cancer’s Staging System for Lung Cancer (32).

For PLELC patients treated with ICI combination therapy, in the first four to six treatment cycles, most of them received a triple-drug regimen including platinum, non-platinum anti-cancer agents, and PD-1/PD-L1 inhibitors. Subsequently, the PD-1/PD-L1 inhibitor was given as maintenance. Two patients were treated with nivolumab plus bevacizumab. One patient received durvalumab combined with tremelimumab. For those treated with ICI monotherapy, they received PD-1/PD-L1 inhibitors for treatment and subsequent maintenance. In our study, PD-1 inhibitors included pembrolizumab, nivolumab, sintilimab, and camrelizumab, and PD-L1 inhibitors included atezolizumab and avelumab.

### Multiple immunohistochemistry

mIHC staining was conducted at the Genecast Biotechnology Co., Ltd. (Beijing, China). For this study, 4-μm thick PLELC tissue sections were fixed in formalin and embedded in paraffin for each panel. The slides were deparaffinized, rehydrated, and subjected to epitope retrieval by boiling at 97°C in tris-EDTA buffer (pH = 9; Klinipath #643901, Duiven, The Netherlands) for 20 min. Endogenous peroxidase was blocked by incubation in Antibody Diluent/Block (PerkinElmer #72424205, Massachusetts, USA) for 10 min, and proteins were subsequently blocked in 0.05% Tween solution containing 0.3% bovine serum albumin for 30 min at room temperature. Levels of PD-1, PD-L1, lymphocyte activation gene 3 (LAG3), T cell immunoglobulin, and mucin domain 3 (TIM3), and CD3 in panel 1 and CD4, CD8, CD68, forkhead box P3 (FOXP3), and cytokeratin (CK) in panel 2 were sequentially detected.

Sections were incubated with primary antibodies against PD-L1 (CST13684, clone E1L3N, 1:100, CST), LAG3 (ab40466, clone 17B4, 1:100, Abcam), TIM3 (CST45208S, clone D5D5R, 1:50, CST), CD3 (ZM-0417, clone LN10, 1:50, Zsbio), CD4 (ZA-0519, clone EP204, 1:100, Zsbio), CD8 (ZA-0508, clone SP16, 1:200, Zsbio), CD68 (ZM-0060, clone KP1, 1:1,000, Zsbio), and CK5/6 (ZM-0313, clone OTI1C7, 1:100, Zsbio), for 1 h at room temperature, and overnight at 4 °C with PD-1 (ZM-0381, clone UMAB199, 1:400, Zsbio), Foxp3 (ab20034, clone 236A/E7, 1:100, Abcam) antibodies. An Opal polymer anti-rabbit/mouse horseradish peroxidase (HRP) Kit (PerkinElmer #2414515, Massachusetts, USA) was used as the secondary antibody, and sections were incubated at 37 °C for 10 min. Tyramine signal amplification (TSA) visualization was then performed using the Opal Seven-color Multiplex Immunohistochemistry Kit (NEL797B001KT, PerkinElmer, Massachusetts, USA), containing fluorophores (DAPI), Opal 520 (CD3, CK5/6), Opal 650 (CD68, TIM3), Opal 620 (LAG3, CD4), Opal 690 (PD-L1, Foxp3), Opal 570 (PD-1, CD8), and TSA Coumarin system (NEL703001KT, PerkinElmer, Massachusetts, USA). After labeling all the five antigens in each panel, microwave treatment was performed using Tris-EDTA buffer (pH = 9; Klinipath #643901, Duiven, the Netherlands) to remove the TSA-antibody complex for 20 min at 97°C. All the slides were counterstained with 4’,6-diamidino-2-phenylindole (DAPI) for 5 min and mounted using the Antifade Mounting Medium (NobleRyder #I0052, Beijing, China) for imaging.

Slides were scanned using the PerkinElmer Vectra system (Vectra 3.0.5; PerkinElmer, Massachusetts, USA). Multispectral images were unmixed with spectral libraries using the inform Advanced Image Analysis software (inForm 2.3.0; PerkinElmer, Massachusetts, USA). For batch analysis, an algorithm was acquired by training 10 to 15 representative multispectral images. Corresponding fluorescent staining intensities of X, 2X, or 3X or more were determined as 1+, 2+, or 3+, respectively, for each biomarker. The scoring formula was H-score = (3+) %×3+ (2+) % × 2+ (1+) %×1 (33).

### Next generation sequencing and mutational analysis

DNA from frozen tumor tissues and paired blood samples or normal tissues was extracted. Gene status was determined by NGS before ICI treatment. Comprehensive genomic profiling of 33 ICI-treated patients was performed by NGS using a 425-, 520-, or 1021 cancer-related gene panel (13 cases with the 1021 panel (Geneplus, Beijing, China), 10 cases with the 425 panel (Geneseeq, Nanjing, China), 5 cases with the 520 panel (Burning Rock, Guangzhou, China), and the other cases with 139 and 168 panel). Sequencing methods have been described previously in detail (34–36).

### Statistical analysis

Responses were assessed according to the Response Evaluation Criteria in Solid Tumors (RECIST) version 1.1 (37, 38). Progression-free survival (PFS) was defined as the time from the initial ICI treatment to the date of disease progression or death due to any cause or the date of the last follow-up (1 August 2021) (38). Overall survival (OS) was calculated from the start of immunotherapy to death or the last follow-up (1 August 2021). Patients with complete response (CR), partial response (PR), or long-term stable disease (SD, lasting over 6 months) were categorized into the durable clinical benefit (DCB) group. The non-durable clinical benefit (NDCB) group comprised patients with disease progression and SD lasting less than 6 months.

The data and figures were described by the median and interquartile range (IQR). A two-sided Mann–Whitney U test was used to compare the expression percentages of different immune markers between the DCB and NDCB groups. The cut-off values of PD-1, PD-L1, and LAG3 expressions were determined using the surv_cutpoint function in the R package, survminer (39). Tumors with immune marker expression over the cut-off value were deemed positive. Survival was plotted by the Kaplan–Meier method, and survival differences were compared by the log-rank test. Statistical analyses were performed using SPSS version 22.0 (SPSS, Inc., Chicago, IL) and R version 3.5.1. In a two-tailed test, a p-value of <0.05 was deemed statistically significant.

## Results

### Clinical characteristics of ICI-treated PLELC patients

Forty-eight metastatic PLELC patients treated with ICI in GLCI between January 2016 and May 2021 were included. The clinicopathological characteristics of these patients are summarized in **Table 1**. As of 1 August 2021, thirteen patients were undergoing ICI treatment and 25 patients (52.1%, 25/48) were alive. The median follow-up time was 45.6 months (range: 3.0–81.0 months).

**Table 1:** Clinical characteristics of the 48 ICI-treated PLELC patients.

Variable	No. of Patients	Percentage
Median age (range)-years	51 (13–73)	
Median TMB	2.3/MB (0.0–26.9)	
**Gender**		
Male	17	35.4
Female	31	64.6
**Smoking**		
Ever	8	16.7
Never	40	83.3
**Stage**		
III	6	12.5
IV	42	87.5
**EBERs**		
1	16	33.3
2 3 NA	8 18 6	16.7 37.5 12.5
**PD-L1 expression** <1% 1–49% ≥50% NA	6 21 14 7	12.5 43.7 29.2 14.6
**Status** Alive Died	25 23	52.1 47.9
**Lines of ICI treatment** First line Second or later line Monotherapy or Combination therapy ICI Monotherapy ICI Combination	23 25 30 18	47.9 52.1 62.5 37.5
**Objective response** PR SD PD	9 33 6	18.8 68.7 12.5
Clinical benefit Durable clinical benefit Non-durable clinical benefit	42 28 14	66.7 33.3

TMB, tumor mutation burden; PR, partial response; SD, stable disease; PD, progression disease; EBERs, EBV encoded small RNAs.

The median age of these patients was 51 years (range: 13–73 years); 64.6% (31/48) were females, and 83.3% (40/48) were non-smokers. Forty-seven patients (97.9%, 47/48) showed good performance status (PS 0–1) before ICI treatment. Twenty-three (47.9%) of these patients were treated with ICI as first-line therapy, and 25 (52.1%) of them received ICI beyond the first line. Thirty patients (62.5%, 30/48) received a single ICI treatment (**Table 1**). All of them were EGFR-negative. Only one patient was ALK-positive but showed primary resistance to crizotinib and nivolumab.

**Figure 1:**
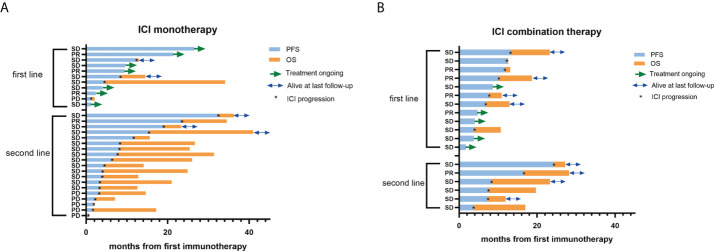
PFS and OS of PLELC treated patients with ICI monotherapy or combination therapy. **(A, B)** Progression-free survival **(**PFS; blue bar) and overall survival (OS; orange bar) (in months) in PLELC patients who underwent treatment with ICI monotherapy or combination therapy at the initiation of first immunotherapy. Best responses during the ICI treatment are shown on the y-axis. First line: first-line treatment with ICI monotherapy or combination therapy. Second line: second- or more line treatment with ICI monotherapy or combination therapy. PR, partial response; SD, stable disease; PD, progression disease. Patients with green arrows were undergoing ICI treatment at the last follow-up. Patients with blue arrows were alive at the last follow-up. *: ICI progression.

Among patients who underwent ICI monotherapy, the objective response rate (ORR) was 13.3% (4/30) and the disease control rate (DCR) was achieved in 80.0% (24/30) of the cases. The median PFS and OS were 7.7 months and 24.9 months, respectively. The one-year PFS rate was 33.3% (**Table 2**, **Figure 1A**). Among patients who underwent ICI combination therapy, ORR reached 27.8% (5/18) and DCR was achieved in 100.0% (18/18) of the cases. The median PFS and OS were 10.1 months and 19.7 months, respectively. The one-year PFS rate was 36.6% (**Table 2**, **Figure 1B**). The ORR was 30.4% among patients who received ICI as the first-line therapy, but only 8.0% among those treated with ICI beyond the first line.

**Table 2:** Clinical responses of the 48 ICI-treated PLELC patients.

Response	Response to ICI			
	Monotherapy(1^st^ line)	Monotherapy(2^nd^+ line)	Combination(1^st^ line)	Combination(2^nd^+ line)
PR	3	1	4	1
SD	7	13	8	5
PD	1	5	0	0
ORR	13.3%		27.8%	
DCR	80.0%		100%	
mPFS	7.7		10.1	
mOS	24.9		19.7	
1-year PFS rate	33.3%		36.6%	

ORR: objective response rate; DCR: disease control rate; mPFS: median progression-free survival; mOS: median overall survival.

### Predictive and prognostic significance of PD-L1 and TMB in PLELC

We first focused on the relationship of PD-L1 expression, TMB, and responses to ICI to examine predictive biomarkers for immunotherapy in PLELC. PD-L1 expression (PD-L1 antibody (22C3)) was detected in 41 pre-treated clinical specimens. In our study, patients with PD-L1 expression ≥1% (TPS score) accounted for 85.4% (35/41) of all cases. A TMB evaluation was available for 33 patients, whereby the median TMB was 2.3/MB (**Table 1**).

**Figure 2:**
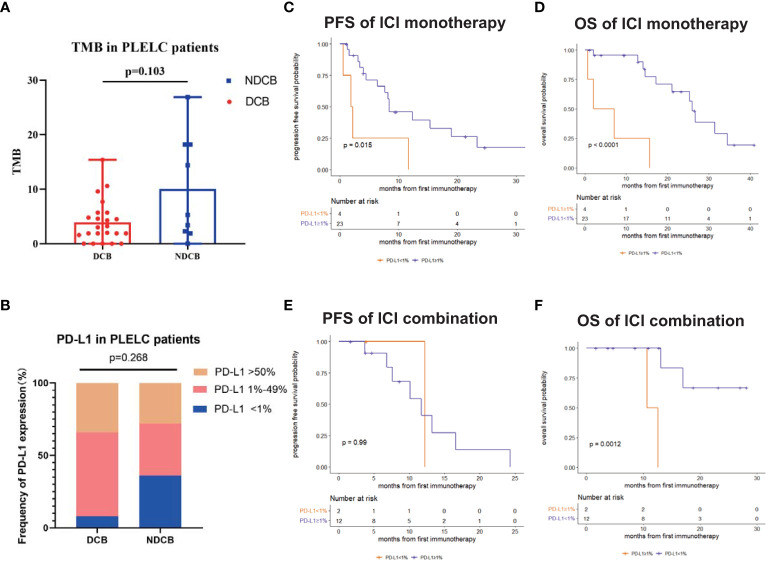
Predictive and prognostic significance of PD-L1 and TMB in PLELC. **(A)** TMB levels in ICI-treated PLELC-DCB patients versus NDCB patients. **(B)** Proportion of different PD-L1 expression in DCB patients versus NDCB patients. **(C, D)** PFS and OS of patients with PD-L1 <1% versus ≥1% in PLELC patients treated with ICI monotherapy. **(E, F)** PFS and OS of patients with PD-L1 <1% versus ≥1% in PLELC patients treated with ICI combination therapy.

Next, we evaluated the differences in PD-L1 expression and TMB between DCB and NDCB patients. No significant differences in TMB were found between DCB and NDCB patients (p = 0.103; **Figure 2A**). Similarly, the proportion of patients with PD-L1 expression <1%, 1%–49%, and ≥50% showed no significant differences between DCB and NDCB groups (p = 0.268; **Figure 2B**).

Survival analysis demonstrated PD-L1 expression as a predictive and prognostic marker for ICI treatment efficacy in PLELC. Patients with PD-L1 ≥1% had a longer PFS (8.4 versus 2.1 months, p = 0.015; **Figure 2C**) and a longer OS (26.0 versus 4.6 months, p <0.001; **Figure 2D**) than those with PD-L1 <1% in the ICI monotherapy group. OS was longer in patients with PD-L1 ≥1% than PD-L1 <1% (NA versus 11.7 months, p = 0.001; **Figure 2F**) who underwent ICI combination therapy. However, PFS showed no statistically significant differences (12.2 versus 11.7 months, p = 0.99; **Figure 2E**).

### Expression of PD-1/PD-L1 and LAG3 correlates with the clinical responses to ICI treatment in PLELC

**Figure 3:**
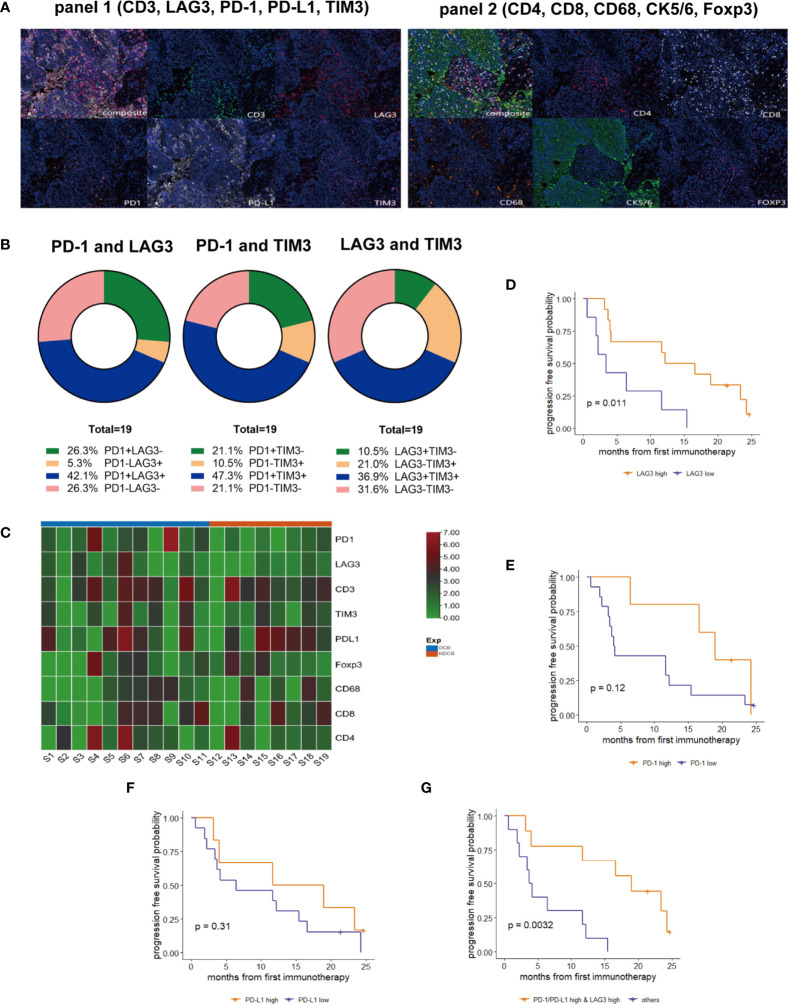
Expressions of PD-1, PD-L1, and LAG3 and their correlation in response to ICI treatment in PLELC. **(A)** Representative multiplexed immunohistochemistry (mIHC) staining for PD-1, PD-L1, LAG3, TIM3, and CD3 (panel 1; left), and CD4, CD8, CD68, FOXP3, and CK (panel 2; right). **(B)** Co-expression of PD-1, LAG3, and TIM3 of mIHC for 19 ICI-treated PLELC patients. **(C)** Heatmap for PD-1, LAG3, CD3, TIM3, PD-L1, Foxp3, CD68, CD8, and CD4 expressions in mIHC in 19 PLELC-DCB and NDCB patients. **(D–G)** PFS in PLELC with high expression of LAG3; PD-1; PD-L1, and PD-1/PD-L1 and LAG3 versus others.

To examine the correlation between the tumor immune microenvironment of PLELC and clinical responses to ICI, mIHC was performed for 19 ICI-treated PLELC (**Figure 3A**) specimens. The expression patterns of the markers are shown in **Supplementary Figure S1A**. With 1% as cut-off value, TIM3, PD-1, PD-L1, LAG3, FOXP3, and CD68 were detected with positivity rates of 57.9%, 68.4%, 68.4%, 47.4%, 73.7%, and 57.9%, respectively, in PLELC specimens (**Supplementary Figure S1A**). Overall, simultaneous co-expression of PD-1, LAG3, and TIM3 was observed in 36.8% of PLELC specimens. PD-1 and LAG3 were co-expressed in 42.1%, PD-1 and TIM3 in 47.3%, and LAG3 and TIM3 in 36.9% of the PLELC specimens (**Figure 3B**, blue chart area).

Next, the correlation between the levels of expression of these markers and responses to ICI treatment was evaluated. The levels of PD-1 and LAG3 expression were higher in DCB compared to the NDCB group (**Figure 3C**). Using the surv_cutpoint function in the R package, survminer, we determined the optimized cut-off values of expressions of PD-1, PD-L1, and LAG3 as 4.113%, 17.376%, and 0.558%, respectively. Patients with expression above the corresponding cut-off value seemed to have high expression of that immune biomarker. A longer PFS in patients with high versus low LAG3 expression was observed (14.4 versus 3.4 months; p = 0.011, **Figure 3D**). A trend towards longer PFS among patients with high PD-1 (19.0 versus 4.1 months, p = 0.12, **Figure 3E**) or PD-L1 (15.4 versus 6.4 months, p = 0.31, **Figure 3F**) was observed. Next, we determined whether a combined score of these three markers provided additional prognostic information. Nine patients showed both high expression of PD-1/PD-L1 and LAG3 (**Supplementary Figure S1B**), with a markedly longer PFS (19.0 versus 3.9 months, p = 0.003; **Figure 3G**) compared with other patients. Additionally, ORR was 44.4% (4/9) in PLELC patients with high expression of PD-1/PD-L1 and LAG3.

### Gene mutation landscape of the 33 ICI-treated PLELC cases

**Figure 4:**
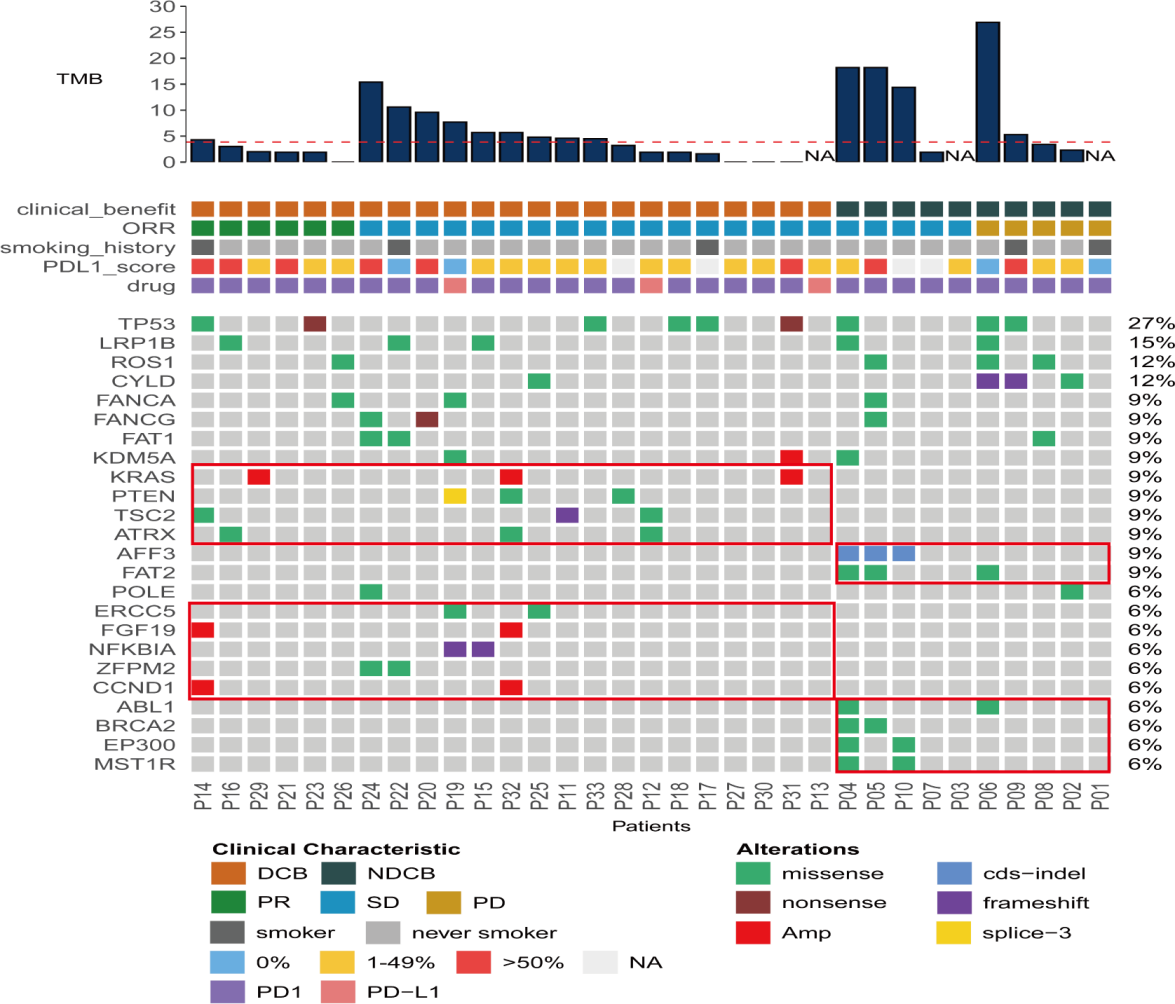
Gene mutation landscape of 33 ICI-treated PLELC patients. Distributions for individual gene mutations in the study cohort as assessed by next-generation sequencing (NGS). Each column represents one patient. Clinical characteristics and NGS-assessed TMB values for each patient are shown on top.

To evaluate the correlation between genic variations and ICI responses in PLELC, NGS sequencing was performed for 33 ICI-treated PLELC patients. Among them, 23 patients were in the DCB, while 10 were in the NDCB groups. No statistically significant differences were obtained between the average TMB of the DCB and NDCB groups (p = 0.1, **Figure 2A**). The top 25 gene variations detected in more than two patients are shown in **Figure 4**. TP53, LRP1B, and ROS1 mutation positivity were detected in 27%, 15%, and 12% of these patients, respectively. The gene alterations that were mutated only in 23 DCB patients were in TSC2, POLE, ZFPM2, NFKBIA, ATRX, ERCC5, and NSD1. Among the NDCB patients, unique gene variations were found in AFF3, FAT2, ABL1, MST1R, and EP300. The differences based on mIHC markers in patients grouped by main genetic alterations showed no statistical significance. The PI3K–AKT pathway was related to a better ICI response in PLELC (**Supplementary Figure S2**), as evidenced by KEGG pathway analysis.

## Discussion

PLELC is a rare and unique subtype of NSCLC. The role of ICI in PLELC remains unclear. Thus far, only a few case reports and small cohort studies have reported on ICI responses in advanced PLELC patients. There is a need to demonstrate the efficacy of ICI for treating advanced PLELC and identify predictive biomarkers of immunotherapy. In this study, we assessed several metastatic PLELC patients who underwent ICI treatment. Our findings confirmed that PLELC patients showed a good response to ICI treatment. Additionally, PD-L1 could predict favorable responses to ICI in PLELC, unlike TMB. mIHC showed that PD-1/PD-L1 combined with LAG3 may serve as a potential marker for predicting ICI responses in PLELC.

Generally, PLELC shows a good response to ICI treatment. Based on our data, ICI monotherapy along with chemotherapy produced satisfactory therapeutic effects for metastatic PLELC. Some patients in our cohort showed a durable response to ICI treatment. The mOS was 24.9 months in the whole GLCI cohort. ORR was achieved at 30.4% at the first line, which was apparently higher than the 8.0% in ICI-treatment beyond the first line in PLELC patients. Thus far, 31 PLELC patients have been treated with ICI according to previous reports (**Table 3**). Fourteen of them underwent ICI monotherapy, while thirteen underwent combination immunotherapy. The median PFS was 15.3 months (**Table 3**, **Supplementary Figure S3**). ORR and DCR were 51.6% (16/31) and 87.1% (27/31), respectively (**Table 3**). In the retrospective cohort from a single institute, including 10 PLELC patients undergoing immunotherapy (ICI group) and 17 undergoing chemotherapy (Chemo group), a longer PFS was observed in the ICI group than in the Chemo group (15.0 and 7.9 months, p = 0.005) (46). These results indicate that ICI may provide a good option for improving the outcome of patients with advanced PLELC. A study showed that ORR was 100% (6/6), extremely high in EBV-positive metastatic gastric carcinoma patients treated with pembrolizumab (23). In EBV-associated nasopharyngeal carcinoma (NPC), the treatment outcome of ICI monotherapy was less than ideal. The ORR was only 25.9% in NPC patients treated with pembrolizumab (25). A significant improvement in PFS was detected in NPC patients treated with toripalimab + chemotherapy (49). It seems that the treatment outcome of ICI varies for different types of EBV-positive malignancies.

**Table 3:** Clinical characteristics of the 31 ICI-treated PLELC patients from previous reports.

ID	Reference	Age	Smoker	PD-L1 expression	ICItreatment	Best response	PFS(m)
1	Narayanan et al. (40)	76	No	TC3/IC3 expression	Atezolizumab	PR	4.2
2	Qiu et al. (41)	56	No	80%	Nivolumab	PR	5.0
3	Kumar et al. (2)	56	Yes	NA	Nivolumab	PR	25+
4	Kumar et al. (2)	37	No	5%	Nivolumab	SD	27+
5	Darrasonet al. (22)	51	Yes	negative	Nivolumab	PR	7
6	Kim et al. (3)	37	No	Positive	Nivolumab	PD	0.3
7	Zhou et al. (42)	NA	NA	90%	Pembrolizumab	SD	12
8	Xie et al. (43)	56	No	30%	Nivolumab + gemcitabine	SD	NA
9	Xie et al. (43)	49	No	60%	NivolumabNivolumab + anlotinib + gemcitabine	SD	5.6+
10	Xie et al. (43)	48	No	15%	Camrelizumab + apatinib	SD	7+
11	Tang et al. (44)	50	No	10%	Nivolumab	PR	5+
12	Chen et al. (45)	41	No	NA	Pembrolizumab + paclitaxel	PR	4.2
13	Fu et al. (46)	68	Yes	80%	Sintilimab	SD	3+
14	Fu et al. (46)	56	No	30%	Pembrolizumab	SD	7
15	Fu et al. (46)	55	No	90%	Pembrolizumab + nab-paclitaxel + carboplatin	PR	9
16	Fu et al. (46)	63	No	70%	Pembrolizumab	PR	14+
17	Fu et al. (46)	70	No	90%	Nivolumab + anlotinib	PR	15+
18	Fu et al. (46)	46	No	60%	Nivolumab +apatinib	PR	17
19	Fu et al. (46)	56	No	80%	Nivolumab + docetaxel	PR	17
20	Fu et al. (46)	61	No	40%	Sintilimab + anlotinib	PR	6+
21	Fu et al. (46)	54	No	NA	Sintilimab + nab-paclitaxel + carboplatin	PR	7+
22	Fu et al. (46)	43	No	80%	Sintilimab	PR	3+
23	Wu et al. (47)	58	No	40%	Sintilimab + anlotinib	PR	8.3+
24	Wu et al. (47)	53	No	30%	Pembrolizumab + nab-paclitaxel	SD	10.9+
25	Wu et al. (47)	48	No	90%	Pembrolizumab	SD	4.2+
26	Wu et al. (47)	56	No	80%	Nivolumab	PR	15.3
27	Wu et al. (47)	63	No	5%	Nivolumab + anlotinib	SD	26.0+
28	Fang et al. (48)	66	No	NA	Anti-PD-1	SD	2.1
29	Fang et al. (48)	50	No	NA	Anti-PD-1	PD	0.9
30	Fang et al. (48)	59	No	NA	Anti-PD-1	PD	2.1
31	Fang et al. (48)	45	Yes	NA	Anti-PD-1	PD	0.9

PD-L1 and TMB are predictive markers of ICI responses in NSCLC (50, 51). However, PD-L1 was a predictive marker for favorable responses to ICI in PLELC. PD-L1 is a marker for adaptive immune responses. Its expression is positively associated with the infiltration of immune cells (29). PD-L1 was highly expressed in PLELC patients, and this is consistent with previous findings, which may explain the good response in ICI-treated PLELC patients (26, 27). The abundance of TILs around tumor lesions in PLELC is well documented. However, with persistent EBV infection, most of the genetic alterations in virus-related cancer do not induce anticancer immune reactions. TMB is not associated with responses to ICI in PLELC. Moreover, TILs tend to transform into an exhausted phenotype, especially the burnt-out CD8+ T cells (52, 53). Moreover, as Chen and colleagues have shown, NSCLC patients with this burnt-out CD8+ T-cell subset show an inferior response to ICI treatment (53). Therefore, although T cells relieve the inhibition of PD-L1 after treatment with ICI, they are not reactive. Moreover, some genes expressed by EBV, including the EBV lytic gene, BILF1, induce internalization and degradation of the major histocompatibility complex (MHC) class I (54). It influences antigen recognition by T cells. Therefore, although most PLELC patients show high levels of PD-L1 expression, some do not show a good response to ICI. The ORR among patients with high PD-L1 expression was 18.8% (9/48). Thus, new predictive biomarkers in PLELC need to be identified.

Findings from mIHC suggested that PD-1/PD-L1 combined with LAG3 could serve as a potential marker for predicting ICI responses in PLELC. In our cohort, PLELC patients with both high PD-1/PD-L1 and LAG3 expression showed a markedly longer PFS compared with others. The ORR was 44.4% (4/9) in these patients. LAG3 and PD-1 are co-expressed in TILs of NSCLC patients (55). Inhibitors of LAG3 can enhance the therapeutic efficacy of PD-1 inhibitors in cell and murine models (55). According to a study comprising 90 ICI-treated patients with advanced NSCLC, those with high LAG3 and low-PD-L1 expression showed markedly lower PFS relative to the low LAG3 and high-PD-L1 expression groups (56). The association of elevated LAG3 expression with insensitivity to PD-1 axis blockade provides evidence for the combination of the LAG3 inhibitor and PD-1 inhibitor. In the phase II clinical trial TACTI-002 (NCT03625323), for NSCLC patients treated with the LAG3 inhibitor IMP321 combined with pembrolizumab, the ORR was 36% (57). Randomized clinical trials in large cohorts are ongoing to demonstrate the effectiveness of the combination of PD-1 and LAG3 inhibitors. In the future, the combination of the PD-1/PD-L1 axis and LAG3 inhibitors may provide another promising choice for PLELC patients. However, the underlying mechanisms need further investigation.

We analyzed the genomic data of ICI-treated patients to examine additional predictive biomarkers for immunotherapy. Similar to the findings of a previous study, the TP53 mutation was the most common genetic alteration in PLELC (58, 59). According to the data from The Cancer Genome Atlas, the TP53 mutation rate in NSCLC is 56.1% (809/1441), higher than that in PLELC (60). Thus, TP53 may not be as important in the development and progression of PLELC as in other types of NSCLC. The PI3K–AKT pathway was related to a good response to ICI in PLELC. Inhibiting the PI3K–AKT pathway can reduce the expression of immunosuppressive cytokines, chemokines, and checkpoint ligands (61). The alteration in the PI3K–AKT pathway may affect immune cell infiltration and cytotoxic functions in PLELC, thus influencing the responses to ICI. Additionally, LRP1B and TSC2 are possible predictive markers for PLELC, and these warrant further investigation (62, 63).

Our study has some limitations, including the retrospective nature and a small number of cases. As it is an observational and retrospective study, some bias may be present in our analyses. Randomized prospective clinical trials with a large cohort are warranted to confirm the efficacy of ICI in PLELC in the future. Additionally, NGS and mIHC to provide integrated data to analyze the genomic landscape and immune biomarkers in PLELC were not performed for all patients. The method of combined PD-1, PD-L1, and LAG3 scoring for mIHC is exploratory and needs further validation in larger and prospective cohorts. We plan to enroll more patients to validate these findings and elucidate the mechanism of favorable responses to ICI in PLELC in the future.

In conclusion, ICI is a promising choice for treating PLELC. On assessing a large cohort of metastatic PLELC patients who underwent ICI treatment, we identified potential predictive markers for immunotherapy. PD-L1 may be both predictive of ICI treatment and prognostic for survival in patients with metastatic PLELC. Moreover, the expression of PD-1/PD-L1 combined with LAG3 may serve as a predictive marker for ICI treatment in metastatic PLELC. In larger prospective trials in the future, we plan to confirm the effectiveness of ICI and identify other potential immune biomarkers for PLELC.

## Data availability statement

No additional raw data is available at this time. Data of this project can be accessed after an approval application to the China National Genebank (CNGB, https://db.cngb.org/cnsa/). Please refer to https://db.cngb.org/search/project/CNP0003388/, or email: CNGBdb@cngb.org for detailed application guidance. The accession code CNP0003388 should be included in the application.

## Ethics statement

Written informed consent was obtained from the individual(s), and minor(s)’ legal guardian/next of kin, for the publication of any potentially identifiable images or data included in this article.

## Author contributions

Y-MZ, KY, and YC contributed equally to this work. X-CZ conceptualized and designed the study. L-LG, W-BZ, and JC collected the clinical data. ZX, Z-YL, S-LZ, D-XL, and H-HZ performed experiments. Y-MZ, KY, and YC analyzed the data and wrote the manuscript. J-JY, X-NY, QZ, B-CW, W-ZZ, H-YT, R-QL, D-KZ, H-BZ, and Y-LW advised on the design of the study and revised the manuscript. All authors contributed to the article and approved the submitted version.

## Funding

This work was supported by following grants: the GDPH Dengfeng Program (Nos. DFJH201903 and KJ012019444 and 8197103306 and 8217113880, X-CZ); the National Natural Science Foundation of China Program (No. 82173202, X-CZ); the Guangdong Provincial Key Lab of Translational Medicine in Lung Cancer (No. 2017B030314120, Y-LW); the Guangdong Provincial Natural Science Program (No. 2019A1515010900, X-CZ).

## Conflict of interest

The authors declare that the research was conducted in the absence of any commercial or financial relationships that could be construed as a potential conflict of interest.

## Publisher’s note

All claims expressed in this article are solely those of the authors and do not necessarily represent those of their affiliated organizations, or those of the publisher, the editors and the reviewers. Any product that may be evaluated in this article, or claim that may be made by its manufacturer, is not guaranteed or endorsed by the publisher.

## Supplementary material

Supplementary Figure 1 The expression pattern of markers in 19 ICI-treated PLELC patients. **(A)**. The expressions of CD3, TIM3, PD-1, PD-L1, LAG3, Foxp3, CD68, CD8, and CD4 in 19 ICI-treated PLELC patients. **(B)**. PLELC patients with high expressions of PD-1/PD-L1 (orange) and LAG3 (blue).

Supplementary Figure 2 Gene Ontology (GO) and KEGG pathway analyses for ICI-treated PLELC DCB patients **(A, B)** and NDCB patients **(C, D)**.

Supplementary Figure 3 PFS of ICI-treated PLELC patients from previous reports.
