# Supplementary figures and images for: PD-1/PD-L1 combined with LAG3 is associated with clinical activity of immune checkpoint inhibitors in metastatic primary pulmonary lymphoepithelioma-like carcinoma
Source: Front Immunol. 2022 Oct 3;13:951817. doi: 10.3389/fimmu.2022.951817 (PMC9574915; doi:10.3389/fimmu.2022.951817)

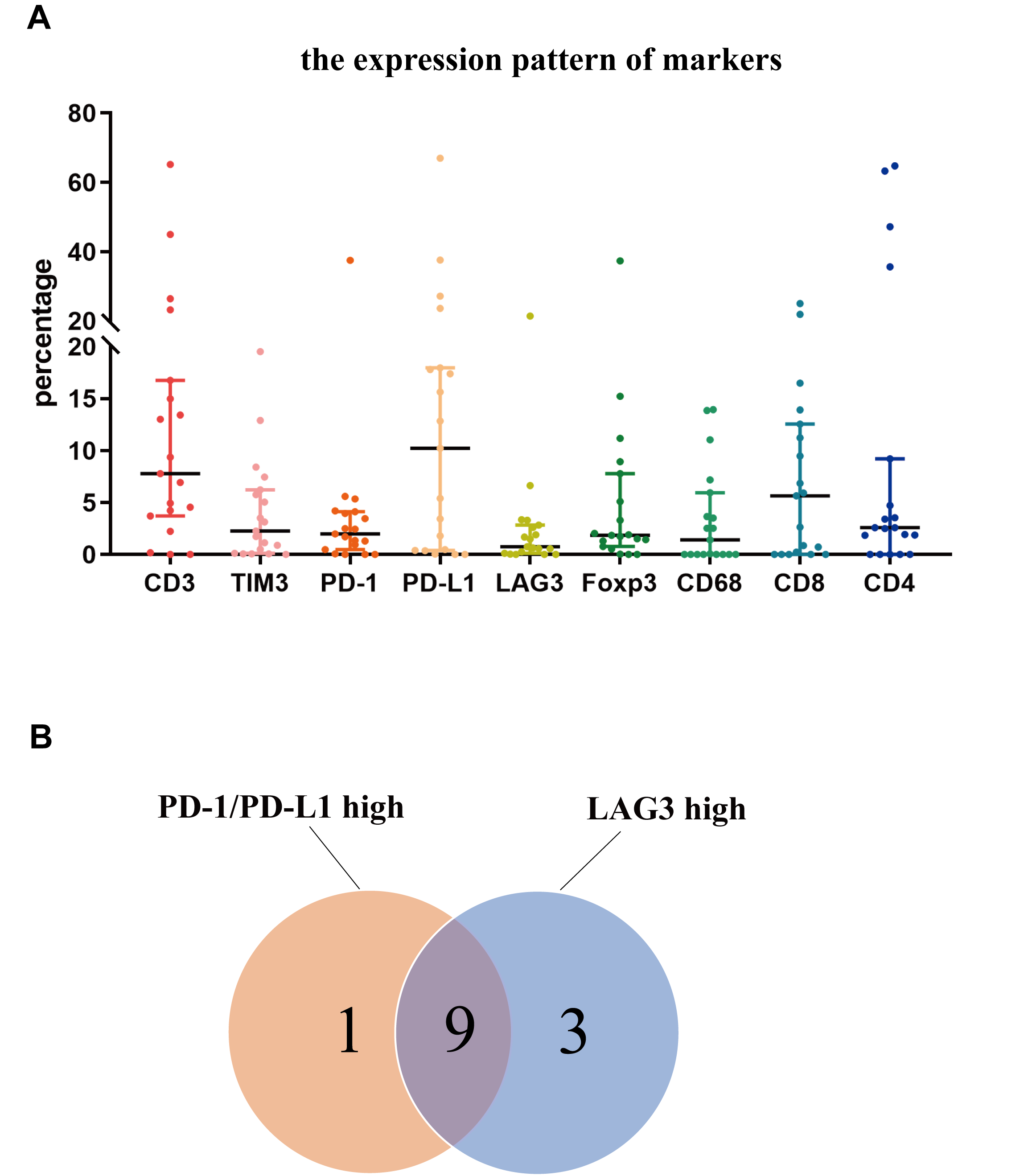

Supplement: Supplementary Figure 1 — The expression pattern of markers in 19 ICI-treated PLELC patients. (A). The expressions of CD3, TIM3, PD-1, PD-L1, LAG3, Foxp3, CD68, CD8, and CD4 in 19 ICI-treated PLELC patients. (B). PLELC patients with high expressions of PD-1/PD-L1 (orange) and LAG3 (blue). [file Image_1.tif]

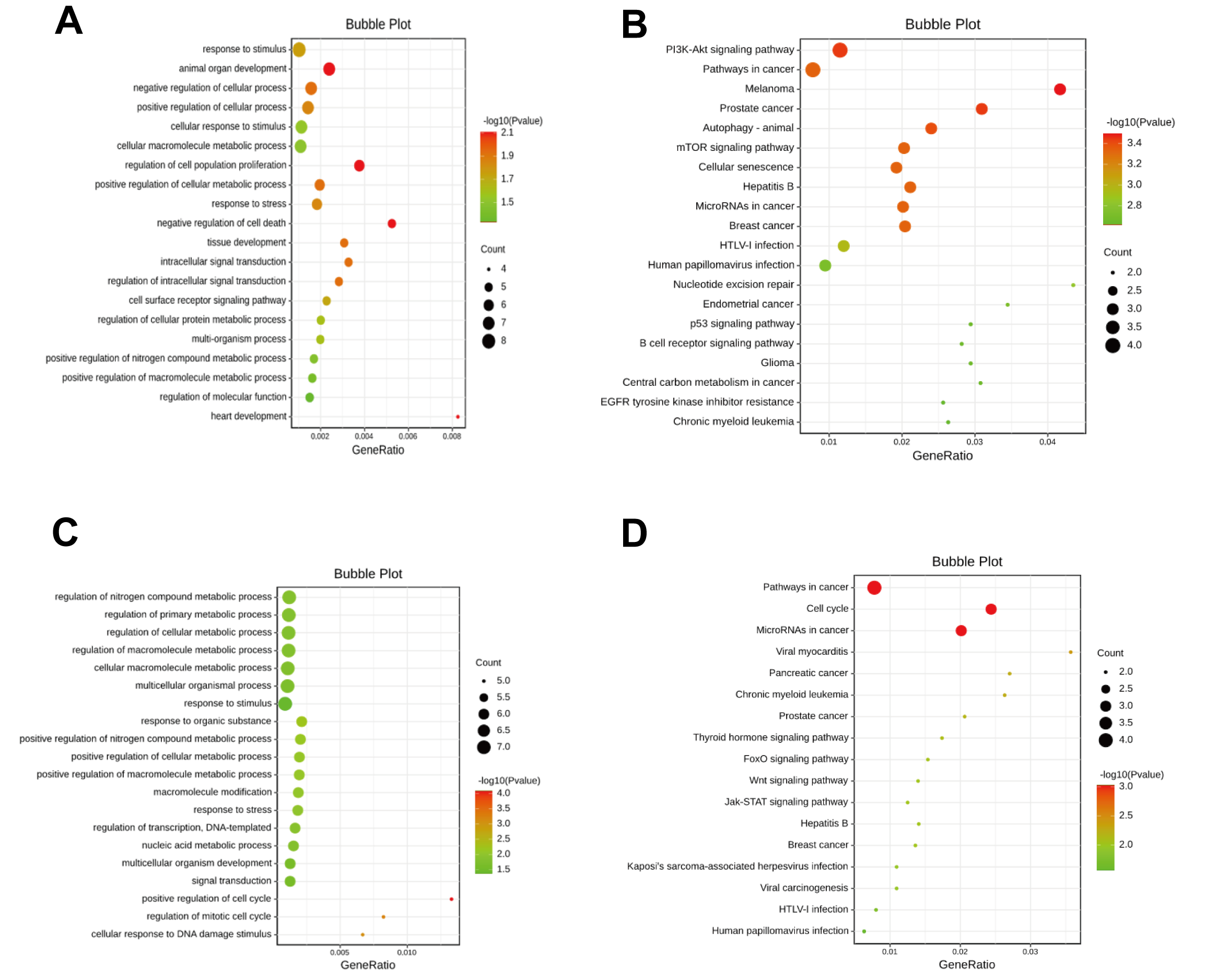

Supplement: Supplementary Figure 2 — Gene Ontology (GO) and KEGG pathway analyses for ICI-treated PLELC DCB patients (A, B) and NDCB patients (C, D). [file Image_2.tif]

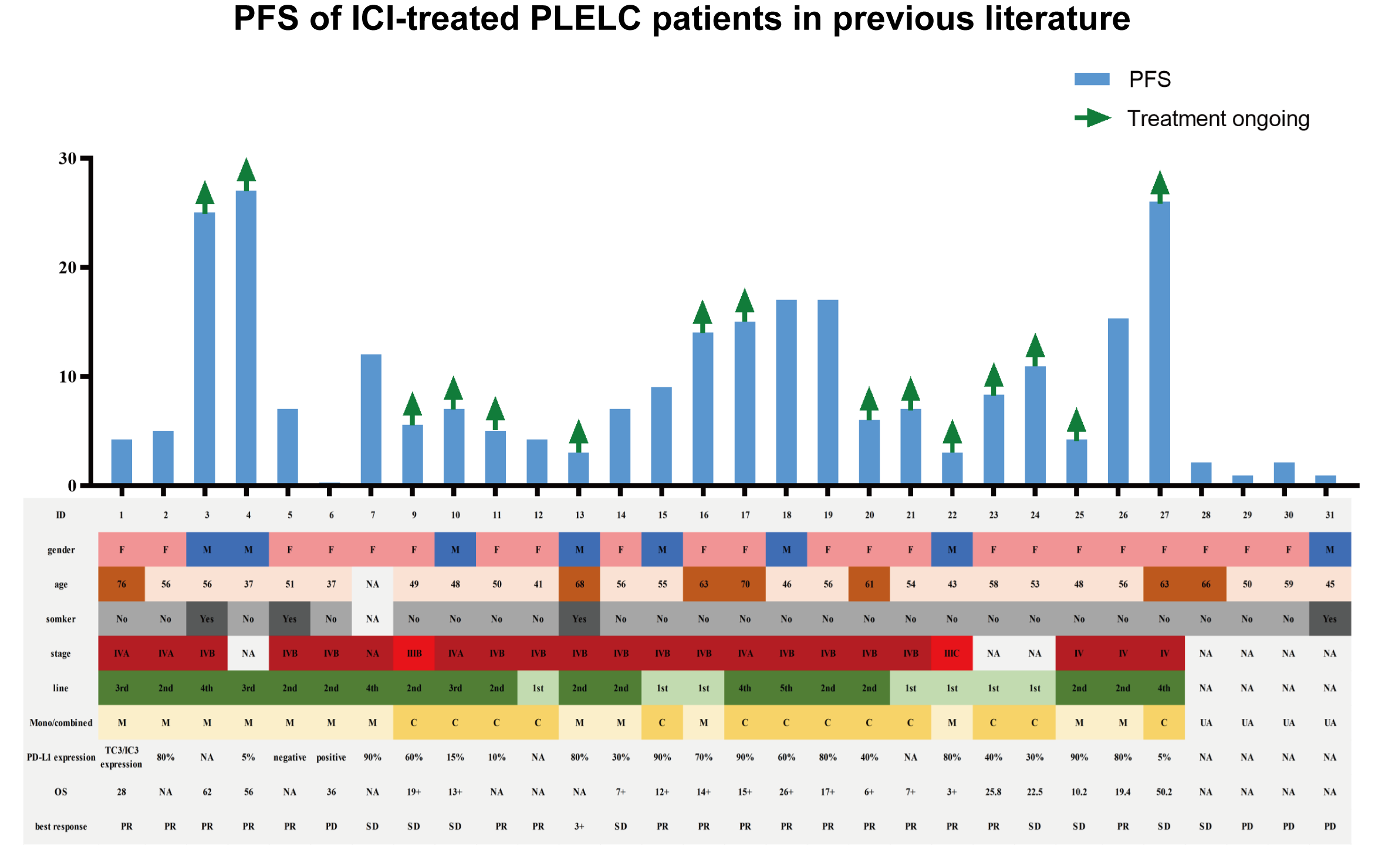

Supplement: Supplementary Figure 3 — PFS of ICI-treated PLELC patients from previous reports. [file Image_3.tif]
